# Time-dependent simvastatin administration enhances doxorubicin toxicity in neuroblastoma

**DOI:** 10.1016/j.toxrep.2020.03.007

**Published:** 2020-04-22

**Authors:** Colin C. Anderson, Meera Khatri, James R. Roede

**Affiliations:** Department of Pharmaceutical Sciences, Skaggs School of Pharmacy and Pharmaceutical Sciences, University of Colorado, Aurora, CO 80045, United States

**Keywords:** Simvastatin, Doxorubicin, Cisplatin, Neuroblastoma, Pleiotropic

## Abstract

Statins have a primary indication for the reduction and management of hypercholesterolemia; however, evidence shows that statins have the ability to increase the toxicity of chemotherapeutics within cancer cells by inducing anti-proliferative, anti-metastatic, and anti-angiogenic effects. More recently, lipophilic statins have shown complex interaction with energy metabolism, specifically acute mitochondrial dysfunction and delayed inhibition of glycolysis. With the goal to demonstrate that statin-mediated enhancement of chemotherapeutics is time-dependent, we hypothesized that the lipophilic statin simvastatin, in conjunction with variable co-exposure of doxorubicin or cisplatin, will enhance the toxicity of these drugs in neuroblastoma. Utilizing human SK-N-AS neuroblastoma cells, we assessed cell proliferation, necrosis, caspase activation, and overall apoptosis of these cells. After determining the toxicity of simvastatin at 48 h post-treatment, 10μM was chosen as the intervention concentration. We found that significant cell death resulted from 1.0μM dose of doxorubicin with 24 h pre-treatment of simvastatin. On the other hand, simvastatin enhancement of cisplatin toxicity was only observed in the co-exposure model. As doxorubicin has strict dosage limits due to its primary off-target toxicity in cardiac muscle, we further compared the effects of this drug combination on rat H9C2 cardiomyoblasts. We found that simvastatin did not enhance doxorubicin toxicity in this cell line. We conclude that simvastatin provides time-dependent sensitization of neuroblastoma cells to doxorubicin toxicity, and our results provide strong argument for the consideration of simvastatin as an adjuvant in doxorubicin-based chemotherapy programs.

## 1 Introduction

Statins are a highly popular class of 3-hydroxy-3-methylglutaryl-CoA (HMG-CoA) reductase (HMGCR) inhibitors for the treatment of hypercholesterolemia [1]. Recently, published studies show the potential of statins as anti-cancer agents via promotion of anti-proliferative effects in certain cancers [2]. Chan et al. report that the primary anti-cancer mechanism involves apoptosis via the depletion of geranylgeranylated proteins as a down-stream effect of mevalonate pathway inhibition. Furthermore, inhibition of geranylgeranyl transferases mimicked the toxicity seen by lovastatin. Simvastatin acts within neuroblastoma through several distinct pathways including inhibition of the mevalonate pathway, depletion of the vital mitochondrial electron transport substrate Coenzyme Q10 (CoQ10), as well as inhibiting the glycosylation/prenylation of important membrane proteins (i.e. dolichol, a product of the HMG-CoA reductase pathway) [3,4]. In the most general sense, inhibiting HMGCR depletes cells of *de novo* cholesterol synthesis that is vital for cancer proliferation. Consequently, statins also deplete byproducts of the mevalonate pathway used for the glycosylation and prenylation of a multitude of membrane proteins. For instance, depletion of the dolichol supply in the cell leads to the inhibition of p-glycoproteins (i.e. ABCB1) and transporter proteins (i.e. GLUT1/4) [3]. Inhibition of these transporters will impair the cell’s ability to detoxify xenobiotics, such as chemotherapeutics. Furthermore, inhibition of glucose transporters (GLUT1-4) could potentially hinder the Warburg phenotype and cause metabolic stress in cancer [5]. In a recent publication, our laboratory reports significant mitochondrial dysfunction following an acute exposure of 50μM simvastatin, a moderate-intensity lipophilic statin, to SK-N-AS cells [6]. This mitochondrial dysfunction is quickly resolved, followed by a delayed dampening of glycolytic activity leading to apoptosis. Because of these observations of altered bioenergetics in neuroblastoma cells shortly after simvastatin exposure, it is our belief that statins may also disturb the delicate energetic balance required for cancer cells to proliferate and metastasize. It should be noted that statins do have off-target adverse effects as well, including the most common adverse reaction of statin-associate muscle symptoms (SAMS) presenting in 10–29 % in patients. However, there has been extensive safety evaluation of these compounds, and they remain an established therapy across the globe [1].

Neuroblastoma is a common pediatric cancer that afflicts 1–3 out of every 100,000 children up to 14 years of age, and develops from a mutation in the differentiation pattern of cells derived from the neural crest [7]. This leads to uncontrolled cell growth and the formation of a solid tumor within the neck, adrenal or retroperitoneal, thoracic, and/or pelvic regions [8]. Because of this solid tumor morphology, anthracycline chemotherapy is the most effective treatment, with doxorubicin (DOX) being one of the most commonly used among this drug category [9]. DOX inhibits cancer growth by intercalating with DNA causing double-stranded breaks and fragmentation of nuclei, as well as by inhibiting RNA polymerase activity [10]. This therapy has been shown to abate genotoxicity in hepatocellular carcinoma rat models, and is often used as a control to compare the efficacy of novel chemotherapeutic agents [11,12]. DOX also induces mitophagy by interfering with the oxidative phosphorylation pathway and producing reactive oxygen species (ROS) that cause DNA damage [13]. As DOX is a mitochondrial toxicant, it has a strong association with cardiotoxicity in the form of cardiomyopathy or congestive heart failure, which limits its administration [14]. For this reason, DOX is most effective in cell types that utilize a large amount of mitochondrial respiration (i.e. cardiac muscle) and has been shown to cause cytotoxicity via iron accumulation in the mitochondria of cardiomyocytes [15,16]. DOX also disrupts calcium transport across the plasma membrane and causes an increase in its permeability, resulting in cellular damage [17]. Due to these cardio-toxic endpoints, there is a threshold amount of DOX administered to a patient before they must be switched to a different treatment. As such, current research mostly focuses on either synthesizing better compounds, or increasing the effectiveness of already tested and proven drugs. Cisplatin (CP), a platinum containing therapeutic, is another common chemotherapeutic in neuroblastoma treatment working by a similar mechanism of DNA intercalation and replication interference. CP specifically binds to purine bases creating DNA strand cross-links [18]. This drug can readily bind with plasma proteins allowing it to penetrate into kidney, liver, colon, small intestine, and testicles contributing to its nonspecific targeting in neuroblastoma [19]. CP binds to DNA and inhibits gene replication and synthesis leading to apoptosis [20]. Although some of this drug filters through the kidneys, majority is retained in tissues of the body for decades after treatment potentially resulting in delayed-onset nephrotoxicity, ototoxicity, and neurotoxicity [18].

By testing these two commonly employed chemotherapeutic agents in conjunction with simvastatin, we hope to see a reduction in their effective dosage *in vitro*, which may translate to enhanced efficacy and a reduction in the associated toxic side effects. Our data presented here shows an enhancement of DOX toxicity when SK-N-AS cells are pretreated for 24 h with simvastatin. Furthermore, similar enhancement of toxicity was not observed in H9C2 rat cardiomyocytes, representing the mitochondrial rich cardiac muscle phenotype. This cell model has been used extensively in research involving mitochondrial toxins, with one study showing slightly decreased respiratory parameters after 24 h exposure to 10 μM simvastatin [21,22].

Interestingly, the simvastatin-CP interaction differed compared to DOX in that only simultaneous co-exposure showed enhanced toxicity. In our report of simvastatin toxicity in SK-N-AS cells, we have defined a bi-phasic hit to the energetics after exposure, which adds another layer of complexity and utility of this potential adjuvant therapy [6]. Due to this, we propose a time-dependent mechanism of simvastatin-mediated enhancement of DOX and CP toxicity in neuroblastoma. Additionally, this interaction may not be as present in off-target tissues, such as cardiomyocytes, allowing for improved targeting and lowered effective dosages.

## 2 Materials and methods

### 2.1 Reagents

All reagents were purchased from Sigma (St. Louis, MO) unless otherwise noted. Simvastatin and DOX stock solutions were prepared in DMSO, while CP was dissolved in saline.

### 2.2 Cell culture

Human neuroblastoma SK-N-AS and rat cardiomyoblast H9C2 cell lines were purchased from ATCC and cultured separately in Dulbecco’s Modified Eagle Medium containing 4.5 g/L glucose, 110 mg/L sodium pyruvate, and L-glutamine in 10 cm cell culture plates. SK-N-AS media was supplemented with 10% FBS and 1% non-essential amino acids while H9C2 media was supplemented with only 10% FBS. Using sterile technique, media was changed every 24 to 48 h or cells were split/harvested at 80–90 % confluency.

### 2.3 IncuCyte S3 live cell analyzer

Simultaneous microscopy and live-cell fluorescence analyses were performed with images acquired every 30–60 min of cells plated and treated in a 96-well culture plate. The IncuCyte software was used to measure confluency using a phase analytical mask. Cytotox Green Reagent (#4633, dead cell stain) and Caspase-3/7 Red Apoptosis Assay Reagent (#4704, cleaved caspase) were used to continuously monitor cell health and viability. Simvastatin, doxorubicin, and cisplatin toxicities were assessed by harvesting SK-N-AS and H9C2 cells using 0.25% Trypsin in EDTA and plated in 96 well plates at 50,000 and 5000 cells per well, respectively. Cells were allowed to reach 80% confluency prior to treatment with varying concentrations of simvastatin. Cells were imaged every 30 min for 48 h treatment period. Co-exposures were performed with 10μM simvastatin administered in conjunction with varying concentrations of doxorubicin and cisplatin. The standard manufacturer’s protocol was used for 0.5μM of Cytotox Green and 1.0μM Caspase-3/7 Red. Analyses were conducted in duplicate with 3–6 wells per treatment.

### 2.4 Flow cytometry

Apoptotic cells were quantified by harvesting SK-N-AS and H9C2 cells using 0.25% Trypsin in EDTA and plated in 12 well plates at 150,000 and 25,000 cells per well. Cells were allowed to reach 80% confluency, then treated with various concentrations of DOX or CP in conjunction with simvastatin and allowed to grow for 48 h. Cells were then harvested and treated with the Annexin V / Dead Cell Reagent (MCH100105) to run on the MUSE® Cell Analyzer (Millipore, Burlington, MA) to obtain live, early apoptotic, late apoptotic, and dead cell counts at >2000 counts per sample. Analyses were run in duplicate batches to total an N = 6 per treatment.

### 2.5 Seahorse XF analysis

Live cell analyses of oxygen consumption rate (OCR) and extracellular acidification rates (ECAR) were measured with the Seahorse XFe96 system (Agilent, Santa Clara, CA). New cell characterization was performed on SK-N-AS cells according to previously published data [23]. New cell characterization was also performed on H9C2 cells to yield an optimum seeding density of 40,000 cells and an FCCP concentration of 2.0μM. SK-N-AS cells were plated at 30,000 cells per well and H9C2 cells were plated at 40,000 cells per well. Both cell types were allowed to seed overnight in a cell culture incubator at 37 °C with an XF cartridge hydrating in deionized water overnight in a non-CO_2_ incubator at 37 °C. On the day of analysis, assay media was prepared similar to culture media (25 mM glucose, 1 mM sodium pyruvate, and 4 mM L-glutamine). The XF culture plate was washed twice with assay media and a final volume of 180 μL assay media was added to cells. The XF culture plate was saturated with calibrant to equilibrate in non-CO_2_ incubator at 37 °C for 30–60 minutes prior to assay initiation.

#### 2.5.1 Acute injections

Real-time analysis allows us to measure baseline respiration of each experimental well prior to exposure for better comparison. Port A on the XF cartridge is designated for acute treatment of control (0.5% DMSO) or 10 μM simvastatin at 20 μL per well. Ports B-D were assigned for each stress test at varying volumes to account for injection into each well (B =22 μL, C = 25 μL, D =27 μL). Simvastatin/DMSO was prepared in assay media at 10X concentration, giving a final well concentration of 10 μM Simvastatin. XF plates were duplicated with an N = 5 per treatment per plate.

#### 2.5.2 Cell energy phenotype

Manufacturer’s protocol was followed for the Cell Energy Phenotype kit with port B containing the FCCP (mitochondrial membrane depolarizer) / oligomycin (ATP-Synthase inhibitor) stressor mix at 2.0μM/2.0μM (final assay concentration).

#### 2.5.3 Cell mito stress

Manufacturer’s protocol was followed for the Cell Mito Stress Test kit with port B containing oligomycin at 2.0μM, port C with 2.0μM FCCP, and port D with a mixture of 0.5μM of each rotenone (complex I inhibitor) and antimycin A (complex III inhibitor) (final well concentration).

#### 2.5.4 Glycolysis stress

Manufacturer’s protocol was followed for the Glycolysis Stress Test kit with port B containing 10 mM glucose, port C with 1.0μM oligomycin, and port D with 50 mM 2-deoxyglucose (2-DG) (competitive hexokinase inhibitor) (final well concentration). NOTE: Assay media for this test does not include glucose or sodium pyruvate.

### 2.6 Statistics

All data sets were analyzed using GraphPad v7 with either a student’s *t*-test (with Welch’s correction where applicable) or one-way or two-way ANOVA with Bonferroni post-hoc testing (unless otherwise noted). * p < 0.05, ** p < 0.01, *** p < 0.001 difference from DMSO (Unless otherwise noted).

## 3 Results

### 3.1 Simvastatin, doxorubicin, and cisplatin show varied toxicity in SK-N-AS neuroblastoma cells

**Fig. 1:**
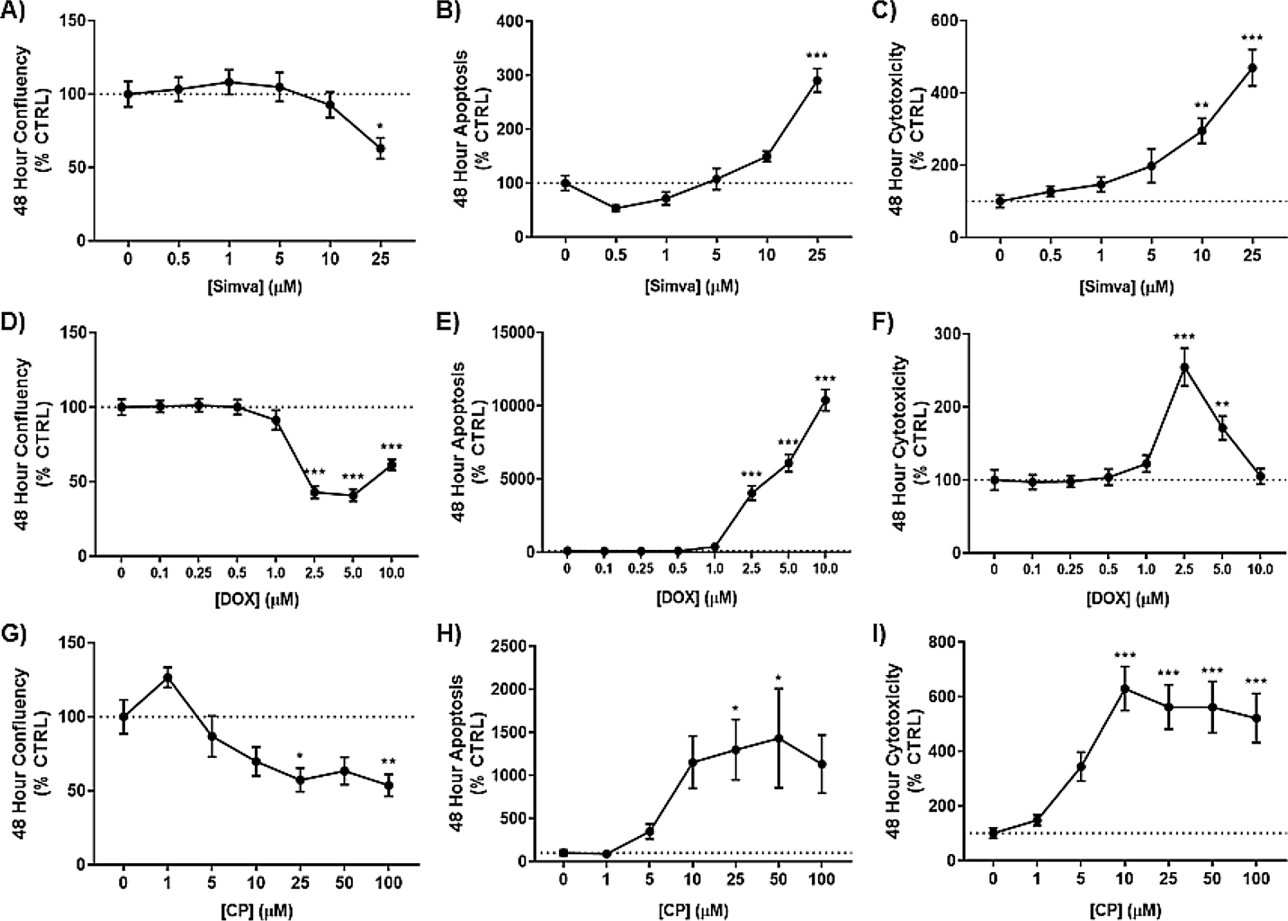
Simvastatin, doxorubicin, and cisplatin show toxicity after 48 h of exposure at various drug concentrations in neuroblastoma. After 48 h exposure to simvastatin (A–C), doxorubicin (D–F), and cisplatin (G–H), the Incucyte Live Cell Analyzer was used to measure confluency (percent)(A, D, G), apoptotic bodies via caspase-3 activation (red fluorescence)(B, E, H), and cytotoxicity with a dead cell stain (green fluorescence)(C, F, I). (N = 6–10, mean ± SEM). (For interpretation of the references to colour in this figure legend, the reader is referred to the web version of this article).

In order to characterize our test compound in regard to their individual toxicity, we first utilized the IncuCyte S3 Live Cell Analyzer system to simultaneously monitor confluency, apoptosis, and necrosis over 48 h after exposure to a range of concentrations of each of our three test compounds: simvastatin, DOX, CP. Expanding on our previously reported observations [6], simvastatin caused significant reductions in cellular proliferation at 48 h (Fig. 1A). Concurrently, simvastatin increased caspase 3/7 activity (Fig. 1B) and dead cell fluorescence (Fig. 1C). DOX induced toxicity in these cells at a much lower concentration compared to simvastatin, with inhibition of proliferation occurring as low as 2.5μM (Fig. 1D). Apoptosis (Fig. 1E) and necrotic cell death (Fig. 1F) were also increased across this range of exposures to DOX. Similarly, CP showed a typical dose-mediated reduction of confluency at 48 h (Fig. 1G). CP did not illicit as strong of an apoptotic response as DOX, yet still had significant increases compared to DMSO treated controls (Fig. 1H). However, CP did cause substantial increases in dead cells over 48 h treatment period in neuroblastoma (Fig. 1I). Therefore, for the remainder of the study a sub-toxic dose of 10μM simvastatin will be used for the DOX and CP co-exposure models.

### 3.2 μM simvastatin causes acute disruption in the energetics of SK-N-AS neuroblastoma cells

**Fig. 2:**
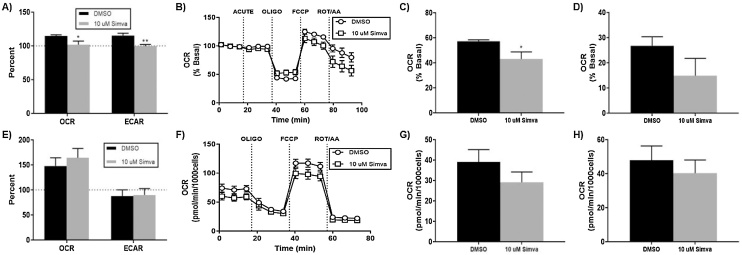
10μM simvastatin causes acute disruptions in mitochondrial respiration, which is mostly resolved at 24 h in neuroblastoma. The Seahorse XF analyzer allows simultaneous monitoring of mitochondrial respiration and extracellular acidification related to glycolysis. After an acute injection of simvastatin, A) metabolic potential was calculated using the cellular energy phenotype assay. B) The cell mitochondrial stress assay after acute injection yielded the measurements for C) ATP production and D) spare respiratory capacity. F) Similar measurements were made after a 24 h exposure for E) metabolic potential, G) ATP production, and H) spare respiratory capacity. (N = 8–10, mean ± SEM).

To assess simvastatin’s contribution to a co-exposure model, we next investigated the effects of simvastatin exposure on energy metabolism as a mechanism to sensitizatize cancer cells to chemotherapeutics. The Seahorse XFe96 platform was employed to evaluate simvastatin-mediated effects on both mitochondrial respiration and glucose utilization in neuroblastoma cells. In our previous report, we observed significant reductions in mitochondrial parameters after acute and 24 -h exposure to 50μM simvastatin [6]. We observed similar deficiencies with an acute exposure to 10μM simvastatin, including reductions in metabolic potential for both respiration and glycolysis (Fig. 2A). The cell mitochondrial stress test (Fig. 2B) revealed simvastatin-mediated reductions in ATP-linked respiration (Fig. 2C) and a slight, but not significant reduction in spare respiratory capacity (Fig. 2D). After 24 h of exposure, these mitochondrial deficiencies were all but resolved within this neuroblastoma system (Fig. 2E–H).

### 3.3 Simvastatin and doxorubicin display enhanced toxicity in Pre-exposure models in SK-N-AS cells

**Fig. 3:**
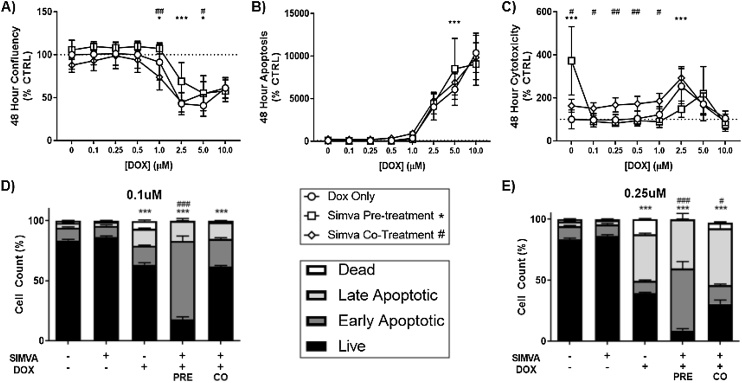
Simvastatin pre-treatment enhances apoptosis, while co-treatment increases cytotoxicity after DOX in neuroblastoma. The Incucyte analyzer was used to observe differences in (A) confluency, (B) caspase activation, and (C) total cell death, at various DOX exposures for 48 h with either a 24 h pre-treatment or co-treatment with 10 μM simvastatin. (N = 10, mean ± SEM). Flow cytometry was used next to confirm apoptosis and cell death using the Annexin V/Dead Cell assay with 10 μM simvastatin pre/co-treatment and 48 h exposure to D) 0.1 μM and E) 0.25 μM DOX. Significant difference in live cells from CTRL (*) and from DOX only (#) is noted. (N = 6–12, mean ± SEM).

To evaluate the combined toxicity of simvastatin and DOX over 48 h, we returned to the IncuCyte platform. For this, we first investigated a 24 -h pre-treatment of 10μM simvastatin prior to exposure to DOX for 48 h. Measurements of confluency, apoptosis (caspase 3/7 activity), and cell death (Cytotox fluorescence) were conducted as previously described, followed by normalization to control. For the pre-exposure model, we observed a slight increase in confluency measurements after 48 h when compared to DOX alone (Fig. 3A). However, the simvastatin pre-treatment did result in an increase in apoptotic cells, but only in the 5.0μM DOX group (Fig. 3B). Assessment of the dead cell stain revealed an initial increase in cell death by the 10μM simvastatin pre-treatment (Fig. 3C); however, this affect is mostly lost with the addition of increasing amounts of DOX. These results, although mechanistically insightful, may not represent the overall effect on a population of cells or tissue. To expand our study, we next confirmed the effects of these exposures on the apoptotic and live cell populations after 48 h using flow cytometry (Fig. 3D–E). Of importance, 10μM simvastatin alone did not result in a significant change in apoptosis or percent live cells compared to the DMSO control. At a dose of 0.1μM DOX, there was a significant decrease in live cells after 48 h post-DOX exposure (Fig. 3D). Additionally, pre-treatment with 10μM simvastatin greatly reduced the percentage of live cells significantly more than DOX alone. Of particular interest, the simvastatin pre-treatment greatly increased the population of cells undergoing early apoptosis by fluorescence associated with Annexin V ((-) statin = 15.7%, (+) statin = 65.4%, ***). This trend of reduced live cell population was replicated in the 0.25μM DOX exposure, where DOX toxicity is enhanced by pre-treatment with 10μM simvastatin (Fig. 3E). Together, the flow cytometry data shows that simvastatin pre-treatment can greatly enhance apoptosis and cell death in neuroblastoma cells treated with DOX *in vitro*.

Interestingly, the different co-exposure protocols produced opposing alterations of proliferation over 48 h, with a decrease in the co-treatment starting at 1.0μM DOX (Fig. 3A). Apoptotic cells associated with DOX generally remained unchanged with both simvastatin exposure models with significant alterations in the pre-treatment only (Fig. 3B). Cell death was consistent with both exposures, as even though the co-treatment is statistically higher than ‘DMSO Only’, this doesn’t seem to be altered by the presence of DOX across the differing lower concentrations (Fig. 3C). These data suggest that the increase in cell death seen in the co-treatment may be due to the acute effect of 10μM simvastatin alone, as shown previously (Fig. 1C).

### 3.4 Simvastatin and cisplatin display enhanced toxicity in Co-exposure models in SK-N-AS cells

**Fig. 4:**
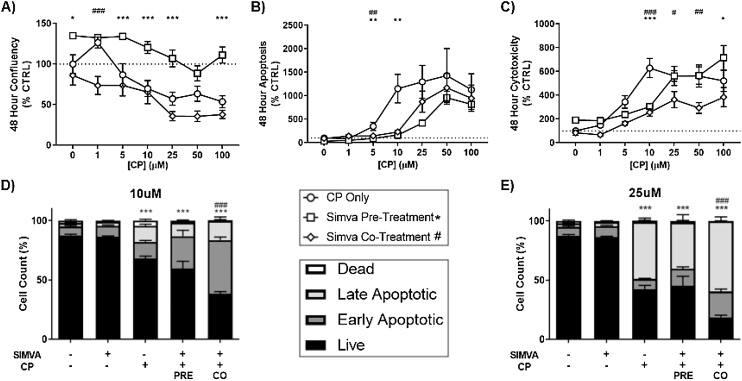
Simvastatin co-treatment enhances cytotoxicity of CP in neuroblastoma. The Incucyte analyzer was used to observe differences in (A) confluency, (B) caspase activation, and (C) total cell death, at various CP exposures for 48 h with either a 24 h pre-treatment or co-treatment with 10 μM simvastatin. (N = 10, mean ± SEM). Flow cytometry was used next to confirm apoptosis and cell death using the Annexin V/Dead Cell assay with 10 μM simvastatin pre/co-treatment and 48 h exposure to D) 10 μM and E) 25 μM CP. Significant difference in live cells from CTRL (*) and from CP only (#) is noted. (N = 6–12, mean ± SEM).

To expand the potential application of simvastatin as an adjuvant therapeutic to common cytotoxic chemotherapeutic agents, we next performed similar IncuCyte analyses with combinations of simvastatin and CP over 48 h (Fig. 4A–C). Similar to DOX, we observed a potential protective effect of simvastatin pre-exposure on proliferation (Fig. 4A). In contrast to the DOX model, there was also a reduction in apoptosis by simvastatin co-exposure with CP (Fig. 4B). Although statistically significant at specific doses of CP, cytotoxicity trends were difficult to interpret across these concentrations of CP (Fig. 4C). Follow-up flow cytometry experiments again confirmed the impact of these treatments on the cell population. At a CP dose of 10μM, live cell population was significantly decreased after 48 h exposure compared to the saline vehicle control (Fig. 4D). However, simvastatin pre-treatment was unable to enhance the CP-associated loss of live cells at both CP concentrations tested (Fig. 4D–E). Surprisingly, Pre-exposure appeared to have a slight, but non-significant protective effect at 25μM CP.

With regard to CP, co-treatment was the most potent protocol for the enhancement of toxicity. Although only significant at one dose, co-treatment trended toward an enhanced loss of confluency across the all doses of CP tested (Fig. 4A). Similar to pre-treatment, co-treatment with simvastatin and CP showed no significant induction of apoptosis when compared to CP alone (Fig. 4B). Simvastatin co-treatment slightly mediated the dead cell signal across the range of CP exposures (Fig. 4C). Flow cytometry analyses of cells co-treated with 10μM simvastatin revealed that the reduction in live cells was greatly enhanced (Fig. 4D). Observed in DOX, simvastatin/CP treatment showed an increase in early apoptosis in the effective treatment scheme ((-) statin = 14.1%, (+) statin = 45.3%, ***). When the CP dose increases to 25μM, the simvastatin Co-exposure maintains its enhancement effect on CP-mediated loss of live cells (Fig. 4E). These data suggest that the addition of simvastatin to both DOX and CP regimes may enhance cytotoxicity, but through differing mechanisms. However, in relation to applications in the clinic, coordinating a precise co-exposure to a patient with drugs intended for repeated administration and established steady state concentrations could prove impossible. With this in mind, we decided to focus on the “Simva + DOX” co-exposure model, as the pre-treatment conditions best represents a single dose of chemotherapeutic on top of steady-state simvastatin regiment.

### 3.5 Doxorubicin shows significant toxicity in H9C2 rat cardiomyocytes, while simvastatin toxicity is diminished

**Fig. 5:**
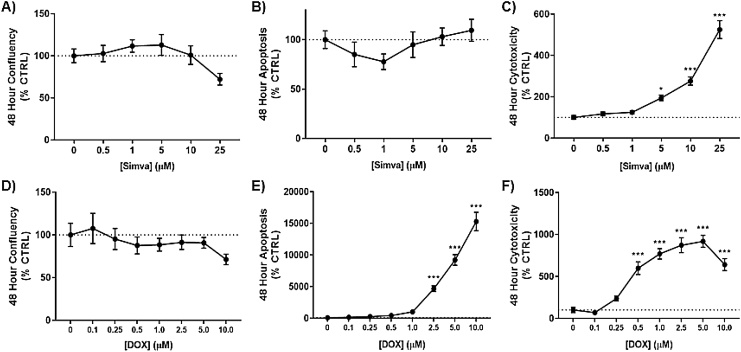
H9C2 rat cardiomyocytes show toxicity with increasing concentrations of simvastatin and DOX. After 48 h exposure to simvastatin (A–C), doxorubicin (D–F), the Incucyte Live Cell Analyzer was used to measure confluency (percent)(A, D), apoptotic bodies via caspase-3 activation (red fluorescence)(B, E), and cytotoxicity with a dead cell stain (green fluorescence)(C, F). (N = 10, mean ± SEM). (For interpretation of the references to colour in this figure legend, the reader is referred to the web version of this article).

As previously mentioned, cardiomyocytes represent an off-target cell population that is affected by DOX treatments. To validate that simvastatin could be a potential and effective adjuvant therapeutic, we next investigated co-exposure models on H9C2 rat cardiomyocytes. IncuCyte analyses of simvastatin toxicity over 48 h revealed similar, yet blunted effects on proliferation and apoptosis compared to SK-N-AS cells (Fig. 5A-B). Simvastatin-mediated depression of proliferation, although not significant in H9C2 cells, followed a similar pattern to SK-N-AS (Fig. 1A). Apoptosis, however, was not induced at any tested concentration of simvastatin after 48 h exposure in H9C2 (Fig. 5B). Surprisingly, dead cell fluorescence was comparable in both cell models, with significant increases at higher concentrations (Fig. 5C).

Whereas DOX caused significant alterations of proliferation, apoptosis, and cell death after 48 h in SK-N-AS neuroblastoma cells starting at 2.5μM (Fig. 1D-F), DOX potency was next evaluated in H9C2, with varied results across the measured parameters (Fig. 5D-F). In these cardiomyocytes, DOX was unable to significantly decrease proliferation (Fig. 5D), yet caspase 3/7 activity was induced as low as 2.5μM (Fig. 5E). Dead cell fluorescence, however, revealed a much lower effective concentration for DOX exposure (Fig. 5F). Through these analyses, we observed cell-specific differences in toxicity for both simvastatin and doxorubicin.

### 3.6 μM simvastatin does not alter cellular energetics in H9C2 rat cardiomyocytes

**Fig. 6:**
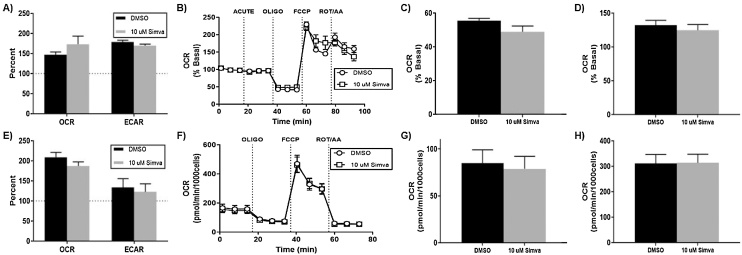
H9C2 rat cardiomyocytes are more resistant to simvastatin-mediated alterations of mitochondrial respiration. A) metabolic potential was calculated using the cellular energy phenotype assay. B) The cell mitochondrial stress assay after acute injection yielded the measurements for C) ATP production and D) spare respiratory capacity. F) Similar measurements were made after a 24 h exposure for E) metabolic potential, G) ATP production, and H) spare respiratory capacity. (N = 8–10, mean ± SEM).

Knowing that cardiomyocytes contain a high concentration of mitochondria for energy production and muscle action, it would be conceivable that equipotent simvastatin exposure would have less affect in this cell model. The Seahorse XF platform was again used to evaluate 10μM simvastatin exposure on H9C2 cells at both acute and 24 -h time-points. The acute phenotype test revealed a small, yet not significant increase in respiratory metabolic potential, with no difference in glycolytic potential (Fig. 6A). The cell mitochondrial stress test showed mostly similar traces, with no apparent differences in ATP-linked respiration or spare respiratory capacity (Fig. 6B–D). At 24 h, there are delayed modifications of metabolic potential in cardiomyocytes (Fig. 6E). The mitochondrial stress test confirmed no alterations in mitochondrial respiratory capacity or energy production (Fig. 6F-H). Observing no significant differences after 10μM simvastatin exposure in this cell line, we confirm that simvastatin has a neuroblastoma-specific potency that is not recapitulated in rat cardiomyocytes.

### 3.7 μM simvastatin does not enhance DOX toxicity in H9C2 cardiomyocytes

**Fig. 7:**
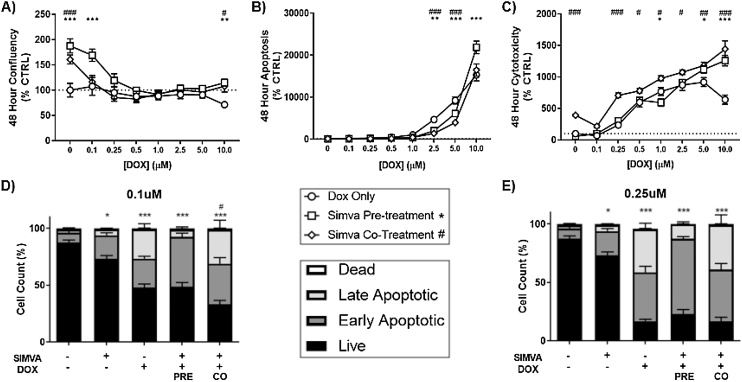
Simvastatin co-exposure does not greatly affect DOX toxicity in H9C2 cells. The Incucyte analyzer was used to observe differences in (A) confluency, (B) caspase activation, and (C) total cell death, at various DOX exposures for 48 h with either a 24 h pre-treatment or co-treatment with 10 μM simvastatin. (N = 10, mean ± SEM). Flow cytometry was used next to confirm apoptosis and cell death using the Annexin V/Dead Cell assay with 10 μM simvastatin pre/co-treatment and 48 h exposure to D) 0.1 μM and E) 0.25 μM DOX. Significant difference in live cells from CTRL (*) and from DOX only (#) is noted. (N = 6, mean ± SEM).

The pre-treatment regiment was applied to H9C2 cells coupled with the IncuCyte analyzer similarly to before. Confluency was only altered at the 0μM and 0.1μM doses of DOX (Fig. 7A), with proliferation being maintained in the presence of simvastatin. Using caspase 3/7 activation as a readout of apoptosis, pre-treatment resulted in less apoptotic cells than DOX alone at 2.5μM and 5μM (Fig. 7B). Remarkably, simvastatin pre-treatment only enhanced cell death at the highest doses of DOX (Fig. 7C). Flow cytometry was performed on the two target concentrations of DOX as used in the neuroblastoma 48 -h co-exposure experiments (Fig. 4). In contrast to SK-N-AS, 10μM simvastatin does cause a significant decrease in live cells in these rat cardiomyocytes (Fig. 7D). However, when combined in pre-treatment, simvastatin does not enhance DOX-mediated loss of live cells at 0.1μM. Nevertheless, an increase in early apoptosis was observed.

For the co-treatment protocol, proliferation was also maintained with simvastatin-only (Fig. 7A), and simvastatin did not greatly alter DOX-mediated loss of confluency. Co-exposure showed less apoptotic cells than DOX alone at 2.5μM and 5μM (Fig. 7B). As for cell death, Co-treatment showed a general and significant increase in cell death caused by acute 10μM simvastatin treatment alone across almost all the DOX exposures (Fig. 7C). Co-treatment did result in slightly higher cell death compared to DOX alone at 0.1μM only (Fig. 7D). Overall, flow cytometry data informs us that DOX has a high potency in the H9C2 cell model, yet simvastatin co-exposure models did not greatly influence toxicity.

## 4 Discussion

The idea of simvastatin as a combination therapy for cancer is not novel; in fact, the vast clinical knowledge of statin safety and effectiveness, added to the popular use and relatively low cost of these drugs, make this an attractive regime. In 2004, Lishner’s group reported on the cytotoxic effect of simvastatin on myeloma cell lines [24]. In a separate report that year, they expanded with co-administration of simvastatin and common chemotherapeutic agents from myeloma *in vitro* [25]. They generalize their main finding to be that simvastatin significantly enhances the cytotoxic agents melphalan and dexamethasone in two myeloma cell lines. In 2010, Dabestan’s group reported a general increase in cell death when DOX and simvastatin were co-administered in HELA cells [26]. An interesting observation in their manuscript includes a pre-treatment model in which the cells were administered DOX or simvastatin before the addition of the other, hinting at a time-dependent interaction. For their HELA cell model, a DOX pre-treatment was more effective than a simvastatin pre-treatment. They also postulate that longer exposures should reveal enhanced apoptosis *in vitro*. More recently, several groups have been combining these compounds across various cancers. One such study identified ‘cell cycle regulator protein RAC1 signaling’ as a critically altered target of combination therapy [27]. However, most evaluations were performed within 24 h of co-exposure, providing only a snapshot of toxic mechanism. Another study investigated combination statin therapy with DOX in aggressive natural killer cell leukemia [28]. This study measured cell growth after 72 h of exposure to several statins and two chemotherapeutics, including DOX, revealing enhancement of DOX-mediated cell death by three statin compounds. Of particular interest, simvastatin was the most effective statin at enhancing DOX toxicity compared to atorvastatin and fluvastatin. Furthermore, Ahmadi et al. recently published a strong review disseminating the drug-drug interactions versus the anti-cancer effects of these statin compounds on chemo-resistance [29]. They concluded that the combination therapy enhances cell death in cancer through both anti-cancer mechanisms, as well as a statin-mediated accumulation of chemotherapeutic within the cells. The lack of mechanistic insight into simvastatin’s exact toxicity across different cancers, as well as off-target tissues, complicates the use of this model in clinical assessments. For instance, simvastatin co-therapy in neuroblastoma is still widely un-researched, with the report by Sieczkowski et al in 2010 being the most recent study [30]. Their manuscript outlines differential apoptosis induction at 24 and 48 h for both simvastatin and atorvastatin. Also, they reported altered DOX accumulation and de-glycosylation of p-glycoproteins within 24 h of 10μM simvastatin. In contrast, our previous study did not show similar alterations in glycosylation of cellular proteins at 24 h [6]. In this current study, we aimed to expand our knowledge of this combination therapy, while focusing on the energetic sensitization by simvastatin as a primary mechanism of action, and the effects of this mechanism on off-target tissue.

The combination analysis of live cell monitoring and end-point flow cytometry has revealed some interesting interactions when inducing cell death in both neuroblastoma and cardiomyocyte cells. First, simvastatin-mediated alterations observed in both cell viability and cellular energetics were more apparent in SK-N-AS neuroblastoma cells compared to H9C2 rat cardiomyocytes. This implies that SK-N-AS are more sensitive to simvastatin toxicity, providing cancer-specific targeting. DOX, on the other hand, showed expected potent toxicity in both cell models, trending with the lack of specificity of this chemotherapeutic. Second, it is important to consider the differences between live cell analysis with simultaneous quantification of fluorescent markers for apoptosis and cell death and the flow cytometry analysis of cell populations after the exposure window. For instance, in the co-exposure studies in SK-N-AS, simvastatin pre-treatment mediated the loss of confluency compared to DOX alone, with significant separation at higher concentrations of DOX (Fig. 3A). However, there were no modifications of DOX-associated caspase 3/7 activation until higher doses of DOX (Fig. 3B). Flow cytometry revealed a strong loss of live cells and a large population of early apoptotic cells associated with the simvastatin pre-treatment (Fig. 3D). At this time-point, cells have been exposed to simvastatin for a total of 72 h (24 -h pretreatment + 48 -h exposure), and we have yet to observe cell viability at this extended exposure. We can presume that the complex timing of our study has missed a complete profile of simvastatin effects; and can only conclude that for this particular dosing scheme, timing is critical for simvastatin to appropriately sensitize neuroblastoma. In fact, hormetic effects of statins have been observed across multiple cell types, which may explain some of the results we report here [31]. Third, it is apparent that significant induction of apoptosis and markers for cell death do not appropriately describe toxic effects on cell populations. Live cell analysis of apoptosis alone would miss the cell death observed in the whole cell population with flow cytometry. We emphasize that our data reports an enhanced reduction of the live cell population and early apoptosis in neuroblastoma, with no such enhancement observed in cardiomyocytes. Specifically at the 0.1μM DOX exposure, we saw over a 3-fold reduction of live cells and an 8-fold increase in early apoptotic cells when combined with the 10μM simvastatin pretreatment. It is conceivable that in human systems, this could equate to a several fold decrease in effective dose of DOX required for chemotherapy. Thus, patients may be able to remain on DOX therapy longer and with less adverse side effects.

We acknowledge that comparing human neuroblastoma to rat cardiomyocytes is not necessarily indicative of human cardiac muscle. Nevertheless, our data advises the need for the next step of research on murine and human clinical models for this therapeutic protocol. We propose that it is the energetic alterations caused by simvastatin that sensitize the energy-hungry Warburg cancer phenotype of neuroblastoma [32]. Our data informs this hypothesis, showing that enhancement of DOX toxicity is time-dependent when considering simvastatin’s effectiveness. Our laboratory previously reported a bi-phasic model of simvastatin toxicity in these neuroblastoma cells, giving further validation of this hypothesis. Although much research has shown mitochondrial disruption by all the compounds tested in this study, it is perhaps the glycolytic disruptions that are the primary culprit for neuroblastoma sensitization to DOX. To compound glucose deficiencies, we observed a reduction in fuel flexibility across the three major fuel sources at 24 -hs, and a specific reduction in glutamine oxidative capacity after 50μM simvastatin. These specific deficiencies in mitochondrial respiration and glucose utilization provide a primed platform for DOX mechanisms leading to the death of these cells. This, in turn, should reduce the amount of DOX administered for the desired effects of tumor reduction and inhibited metastasis.

In conclusion, we report that simvastatin co-administration in both DOX and CP exposures result in a time-dependent sensitization of neuroblastoma cells to chemotherapeutic toxicities. We propose that this sensitization is centered around statin-induced metabolic stress and alterations in glucose and glutamine metabolism. Combined with the current body of literature, our data supports the advancement of simvastatin co-treatment to investigation in murine models and potentially human clinical trials.

## CRediT authorship contribution statement

**Colin C. Anderson:** Conceptualization, Investigation, Methodology, Validation, Formal analysis, Writing - original draft, Writing - review & editing, Visualization. **Meera Khatri:** Investigation, Formal analysis, Writing - original draft. **James R. Roede:** Conceptualization, Resources, Formal analysis, Writing - original draft, Writing - review & editing, Project administration.

## Declaration of Competing Interest

The authors declare that they have no known competing financial interests or personal relationships that could have appeared to influence the work reported in this paper.

## References

[1] Adhyaru B.B., Jacobson T.A. (2018). Safety and efficacy of statin therapy. Nat. Rev. Cardiol..

[2] Chan K.K.W., Oza A.M., Siu L.L. (2003). The statins as anticancer agents. Clin. Cancer Res..

[3] Atil B. (2016). In vitro and in vivo downregulation of the ATP binding cassette transporter B1 by the HMG-CoA reductase inhibitor simvastatin. Naunyn Schmiedebergs Arch. Pharmacol..

[4] Alizadeh J. (2017). Mevalonate cascade inhibition by simvastatin induces the intrinsic apoptosis pathway via depletion of isoprenoids in tumor cells. Sci. Rep..

[5] Adekola K., Rosen S.T., Shanmugam M. (2012). Glucose transporters in cancer metabolism. Curr. Opin. Oncol..

[6] Kuzyk C.L., Anderson C.C., Roede J.R. (2020). Simvastatin induces delayed apoptosis through disruption of Glycolysis and mitochondrial impairment in neuroblastoma cells. Clin. Transl. Sci..

[7] Schwab M. (2003). Neuroblastoma: biology and molecular and chromosomal pathology. Lancet Oncol..

[8] Tsubota S., Kadomatsu K. (2018). Origin and initiation mechanisms of neuroblastoma. Cell Tissue Res..

[9] Koleini N., Kardami E. (2017). Autophagy and mitophagy in the context of doxorubicin-induced cardiotoxicity. Oncotarget.

[10] Meredith A.M., Dass C.R. (2016). Increasing role of the cancer chemotherapeutic doxorubicin in cellular metabolism. J. Pharm. Pharmacol..

[11] Abd El-Moneim O.M., Abd El-Rahim A.H., Hafiz N.A. (2018). Evaluation of selenium nanoparticles and doxorubicin effect against hepatocellular carcinoma rat model cytogenetic toxicity and DNA damage. Toxicol. Rep..

[12] Burci L.M. (2019). Acute and subacute (28 days) toxicity, hemolytic and cytotoxic effect of Artocarpus heterophyllus seed extracts. Toxicol. Rep..

[13] Bian X. (2001). NF-kappa B activation mediates doxorubicin-induced cell death in N-type neuroblastoma cells. J. Biol. Chem..

[14] Zhang Y.W. (2009). Cardiomyocyte death in doxorubicin-induced cardiotoxicity. Arch Immunol Ther Exp (Warsz).

[15] Ichikawa Y. (2014). Cardiotoxicity of doxorubicin is mediated through mitochondrial iron accumulation. J. Clin. Invest..

[16] Bartlett J.J., Trivedi P.C., Pulinilkunnil T. (2017). Autophagic dysregulation in doxorubicin cardiomyopathy. J. Mol. Cell. Cardiol..

[17] Khalilzadeh M. (2018). Protective effects of magnesium sulfate against doxorubicin induced cardiotoxicity in rats. Life Sci..

[18] Ruggiero A. (2013). Platinum compounds in children with cancer: toxicity and clinical management. Anticancer Drugs.

[19] Chemotherapy with Platinum Compounds: Current Status and Future Directions (1994). Volume 3: dosing, head and neck Cancer, genitourinary cancer, pediatric cancer, tumor of unknown origin, dose intensity, future directions. Kona, Hawaii, February 24-28, 1993. Semin. Oncol..

[20] Cece R., Barajon I., Tredici G. (1995). Cisplatin induces apoptosis in SH-SY5Y human neuroblastoma cell line. Anticancer Res..

[21] Bonifacio A. (2016). Simvastatin induces mitochondrial dysfunction and increased atrogin-1 expression in H9c2 cardiomyocytes and mice in vivo. Arch. Toxicol..

[22] Pointon A. (2013). Phenotypic profiling of structural cardiotoxins in vitro reveals dependency on multiple mechanisms of toxicity. Toxicol. Sci..

[23] Anderson C.C. (2018). Acute maneb exposure significantly alters both glycolysis and mitochondrial function in neuroblastoma cells. Toxicol. Sci..

[24] Gronich N. (2004). Simvastatin induces death of multiple myeloma cell lines. J. Investig. Med..

[25] Drucker L. (2004). Co-administration of simvastatin and cytotoxic drugs is advantageous in myeloma cell lines. Anticancer Drugs.

[26] Sadeghi-Aliabadi H., Minaiyan M., Dabestan A. (2010). Cytotoxic evaluation of doxorubicin in combination with simvastatin against human cancer cells. Res. Pharm. Sci..

[27] Buranrat B., Suwannaloet W., Naowaboot J. (2017). Simvastatin potentiates doxorubicin activity against MCF-7 breast cancer cells. Oncol. Lett..

[28] Henslee A.B., Steele T.A. (2018). Combination statin and chemotherapy inhibits proliferation and cytotoxicity of an aggressive natural killer cell leukemia. Biomark. Res..

[29] Ahmadi Y., Karimian R., Panahi Y. (2018). Effects of statins on the chemoresistance-The antagonistic drug-drug interactions versus the anti-cancer effects. Biomed. Pharmacother..

[30] Sieczkowski E. (2010). Double impact on p-glycoprotein by statins enhances doxorubicin cytotoxicity in human neuroblastoma cells. Int. J. Cancer.

[31] Liao J.K. (2012). Mitohormesis: another pleiotropic effect of statins?. Eur. Heart J..

[32] Aminzadeh S. (2015). Energy metabolism in neuroblastoma and Wilms tumor. Transl. Pediatr..

